# Mechanisms of Strong Hearts, Healthy Communities-2.0 Effects on Weight: A Mediation Analysis

**DOI:** 10.1016/j.jneb.2025.12.008

**Published:** 2026-02-06

**Authors:** Chad D. Rethorst, Margaret Demment, Meredith L. Graham, Seungyeon Ha, Alexandra L. MacMillan Uribe, Brian K. Lo, Jacob Szeszulski, Phrashiah Githinji, David Strogatz, Sara C. Folta, Miriam E. Nelson, Rebecca A. Seguin-Fowler

**Affiliations:** 1 Institute for Advancing Health through Agriculture, Texas A&M Agrilife Research, Dallas, TX; 2 Statistical Consultation Center, Texas A&M University, College Station, TX; 3 Department of Family Relations and Applied Nutrition, University of Guelph, Guelph, Ontario, Canada; 4 Bassett Research Institute, Cooperstown, NY; 5 Friedman School of Nutrition Science and Policy, Tufts University, Boston, MA; 6 Institute for Advancing Health through Agriculture, Texas A&M AgriLife Research, College Station, TX; 7 Texas A&M Agrilife Research Center - Dallas, Dallas, TX; 8 Departmenent of Nutrition, Texas A&M University, College Station, TX; 9 Department of Health & Kinesioglogy, University of Utah, Salt Lake City, UT

**Keywords:** nutrition, physical activity, eating behavior, intervention, women

## Abstract

**Objective:**

To evaluate potential mediators of weight loss in the Strong Hearts, Health Communities-2.0 (SHHC-2.0) trial.

**Design:**

Community-randomized trial (intervention vs delayed intervention). Outcomes were evaluated at baseline and postintervention.

**Setting:**

Eleven rural, medically-underserved communities

**Participants:**

Women (n = 182), mean age 57.2 years, 97.6% White, non-Hispanic.

**Intervention:**

Classes delivered 2 times/wk for 24 weeks, targeting diet and physical activity behaviors.

**Main Outcomes Measures:**

Dependent variable: weight; mediators: diet and physical activity behaviors, and related psychosocial factors.

**Analysis:**

Mixed linear regressions evaluated the effect of mediators on weight loss.

**Results:**

Significant mediators included dietary behaviors (Rapid Eating Assessment for Participants-Short; 31.8%; *P* = 0.03), healthy eating attitudes (23.0%; *P* = 0.03), and dietary cognitive restraint (29.8%; *P* = 0.01). Physical activity did not mediate weight loss; however, a worsening in attitudes toward exercise was a mediator of weight loss (22.6%; *P* = 0.01). Social support for diet and physical activity was unchanged in the intervention group and did not mediate weight loss effects.

**Conclusions and implications:**

Healthy eating attitudes and dietary cognitive restraint represent important targets for future behavioral interventions for weight loss. Hypothesized mediators found to be nonsignificant (i.e., social support) represent opportunities for future intervention optimization.

## INTRODUCTION

Women living in rural areas of the US have higher rates of obesity, lower levels of physical activity, and poorer diet quality compared with their nonrural counterparts.^1,2^ Applying a socioecological perspective to the health disparities experienced by women living in rural settings suggests the need for interventions tailored to the specific individual and contextual needs of this population.^3^ Significant differences in individual dietary and physical activity behaviors, interpersonal factors such as social support, and environmental factors have been identified in rural populations that contribute to increased obesity risk.^4^ Calls to reduce the health gaps between rural and nonrural populations^5^ have resulted in many studies evaluating weight loss interventions targeting adults living in rural areas. Dixon et al.^4^ recently reviewed 18 weight loss interventions conducted in rural locations in the US; while statistically significant weight loss was observed overall, weight loss ranged widely (from 0.2 kg to 11.6 kg), with 11 studies demonstrating clinically-significant mean weight loss. Similarly, a meta-analysis by Porter et al.^6^ on the effectiveness of 50 behavioral weight loss interventions in Australia, Canada, and the US concluded that weight loss interventions conducted in rural settings resulted in a statistically significant mean weight loss of 1.8 kg. However, fewer than half of the participants achieved a clinically-significant reduction in weight, suggesting opportunities for improvement in intervention effectiveness.

Effective interventions for weight loss are particularly needed for women in midlife and older age. On average, women gain approximately 0.5 kg per year during this timeframe, with similar weight gain during perimenopausal and menopausal periods.^7,8^ Furthermore, research has demonstrated postmenopausal increases in adiposity and increased cardiovascular disease risk.^9^ As a result, a scientific statement from the American Heart Association highlights this time of life as an important period for cardiovascular disease prevention and notes the potential for lifestyle interventions to reduce cardiovascular disease risk.^10^ While hormonal changes in women in midlife and older may contribute to weight gain, previous research indicates that weight loss is achievable through lifestyle interventions in this population.^11–13^

The collective evidence, described above, suggests that behavioral interventions, many built from evidence-based recommendations and theory, can lead to weight loss in rural populations and among women in midlife and older; however, refinements to these interventions are necessary to optimize outcomes. Intervention development should be considered an iterative process by which data collected through mixed-methods evaluations are used to identify suboptimal results and inform refinements to the intervention.^14,15^ One approach is to evaluate the mechanisms by which an intervention leads to weight loss. Mediation analysis can be useful to test the proposed mechanisms by which an intervention exerts its effect on both behavioral outcomes and, ultimately, the desired health outcomes. Previous meta-analyses of weight loss interventions have identified behavioral (i.e., improved diet quality, increased fruit/vegetable consumption, reduced energy intake, and increased physical activity) and psychosocial mediators (cognitive restraint, self-efficacy, and self-regulation skills) of weight loss.^16,17^ However, mediation analysis is rarely employed within rural weight loss intervention studies. Among the 50 trials reviewed by Porter et al., 10 (20%) reported mediators.^6^ Without a greater understanding of the mechanisms by which interventions exert their effect, efforts to optimize intervention effectiveness, adapt them for different settings and contexts, scale up and disseminate them more broadly will continue to be limited.

The Strong Hearts, Healthy Communities-2.0 (SHHC-2.0) study, which enrolled women living in rural areas with overweight or obesity, evaluated a multilevel behavior change intervention that was developed and refined through community input specifically for delivery in rural settings. The primary outcome was change in body weight, with an overarching objective to target cardiovascular disease prevention through healthy lifestyle changes (e.g., diet, exercise). Study findings related to process evaluation^18^ and mediation analysis for behavior change from the first trial, SHHC, have been published elsewhere.^19^ The second trial, SHHC-2.0, demonstrated significant improvements in the primary outcome, body weight (mean difference −3.15 kg [95% confidence interval, −4.98 to −1.32]), additional clinical and functional markers, including the American Heart Association’s Simple 7 composite score, and improvements in diet and physical activity behaviors.^20–23^ The purpose of this paper was to conduct an exploratory analysis of how potential diet and physical activity mediators, tied to the overall goals and behavioral aims of SHHC-2.0, were related to weight loss in study participants. We hypothesized that the weight loss observed in participants in the intervention group compared with participants in the comparison group would be mediated by improvements in diet and physical activity behaviors and related psychosocial factors.

## METHODS

We conducted a community-randomized trial between January 2017 and August 2018. Eleven rural, medically-underserved communities in New York were pair-matched, based on Rural-Urban Commuting Area code and population size. A statistician not affiliated with the study randomized the communities to either the SHHC-2.0 intervention or a delayed-intervention comparison condition, in which intervention delivery followed the 24-week primary outcome period. Methodological details relevant to this study are below; a full description of the study design and related reports is published elsewhere.^20–24^ The study was approved through a full board review by Cornell University (Institutional Review Board no. 1402004505) and Bassett Medical Center Institutional Review Boards. Before baseline data collection, participants provided written informed consent.

### Study Sample

Participants were female and aged > 40 years at enrollment, and were required to have a body mass index (BMI) > 30, or a BMI 25–30 plus < 1 weekly occurrence of 30 minutes of leisure physical activity. Exclusion criteria were (1) systolic blood pressure > 160 mmHg or diastolic blood pressure >100 mmHg, (2) resting heart rate <60 or >100 beats per minute, (3) cognitive impairment, (4) current or planned participation in another health behavior change program in the next 12 months, (5) inability to obtain permission from a health care provider for participation, or (6) unwilling to provide informed consent or to be randomized to either group. Intervention leaders in each community were responsible for recruitment of participants and used a variety of recruitment methods, including social media, radio/newspaper advertisements, flyers, and direct mail postcards.

### Intervention

The SHHC-2.0 intervention included 2 60-minute in-person classes per week for 24 weeks, led by local health educators who were demographically similar to study participants. Intervention leaders attended a 1-day training on the delivery of the SHHC-2.0 intervention and another half-day session on research methods. Leaders also attended weekly support calls during intervention delivery.^24^ Class duration and frequency were determined by formative focus groups conducted by the researchers. Each class covered topics around goals and behavioral aims for both diet and physical activity (Table 1). The SHHC-2.0 intervention aimed to target factors across the individual social and environmental levels of the socioecological model^3,25^ and intervention strategies were informed by Social Cognitive Theory.^26^ Intervention leaders completed fidelity checklists following each class session.^27^

For diet, the goal was to improve dietary patterns, and behavioral aims were informed by US dietary guidelines, Dietary Approaches to Stop Hypertension (DASH), and Mediterranean dietary patterns.^28–31^ The specific behavioral aims are as follows: decrease energy intake, increase fruit and vegetables, increase whole grains, decrease saturated and trans fats, decrease processed foods, decrease desserts, decrease sugar-sweetened beverages, and decrease sodium. These were targeted through nutrition education lessons, food demonstrations, group discussions, goal setting, and home and grocery store experiences.

For physical activity, the goal was to be as physically active as possible every day; the specific behavioral aims were as follows: increase strength training to ≥ 2 times a week, increase aerobic exercise to at least 5 d/wk, and minimize sedentary time. Additionally, 45 of the 48 classes included progressive strength training and aerobic exercise, which averaged 13 minutes of strength training per class and 24 minutes of aerobic exercise per class. Participants were also encouraged to engage in physical activity outside of class time.

Participants received a participant guide that included foundational knowledge on physical activity and nutrition, class-by-class resources (e.g., exercise and nutrition handouts, websites, and articles), recipes, meal planning guides, and weekly homework (e.g., strength training and aerobic exercise outside class time). Participants were provided with a Fitbit Charge HR, a Withings body scale, exercise DVDs, and a health journal to record monthly goals, weekly food and activity plans, daily food intake, and monthly reflections.

### Measures

At baseline and end of intervention (24 weeks), participants attended a data collection visit that included weight, functional fitness tests, and a survey of self-reported behaviors and psychosocial variables. During designated periods before and at the end of the intervention, data collection also included automated self-administered 24-hour dietary recalls (ASA24)^32^ and accelerometry wear to measure physical activity. Participants were compensated $20 for completion of baseline assessments and $40 at the Week 24 time point. Basic demographic information was collected at baseline. Race and ethnicity were self-reported by participants from the following questions: (1) Which race best describes you? (Check all that apply) American Indian or Alaskan Native, Asian, Black or African American, White, Native Hawaiian and other Pacific Islander, Other race (specify), and (2) Are you of Hispanic or Latino origin? (Yes or No).

#### Primary outcome.

The primary outcome for the study and the present analysis was body weight. Weight was collected using an Omron HBF-510W scale and was measured twice. If the 2 measurements differed by less than 0.1kg, the 2 measures were averaged. If the 2 measurements differed by > 0.1 kg, a third measurement was taken, and the 3 measures were averaged.

#### Mediators.

The measures used to capture potential mediation were selected *a priori* to the analysis based on the intervention goals and behavioral aims (Table 1).

#### Dietary: Overall patterns.

We assessed dietary patterns in 3 different ways. We asked participants to complete 5 self-administered 24-hour dietary recalls using the ASA24 at each assessment time point.^32^ First, the Healthy Eating Index-2015 (HEI) total score and 13 HEI component scores were computed from the ASA24 24-hour dietary recalls using the National Cancer Institute’s simple HEI scoring algorithm, SAS program, and macro^33^ and processed using the National Cancer Institute’s recommendations.^34^ Dietary intake was averaged across participants with at least 2 recalls. We excluded recall data if (1) the response was marked as complete and reported ≤ 100 kcals, (2) the response was marked incomplete and reported < 500 kcals, and (3) the recall reported ≥ 6,600 kcals.^35^ Previously reported results demonstrated no difference in HEI score or energy intake when comparing weekdays to weekend days.^23^ The HEI total and component scores for each participant were averaged at baseline and the end of the intervention. Second, the percentage of total energy intake from ultraprocessed foods was computed using food codes from the ASA24 dietary recalls and the NOVA classification system and code.^36^ Ultraprocessed food categorization according to NOVA has demonstrated convergent validity with calculations of added sugars and macronutrients, and micronutrients.^36^ Finally, the Rapid Eating Assessment for Participants-Short Version (REAP-S) (score 13–39), a validated 16-item scale for assessing dietary quality.^37^

#### Dietary: Nutrient intake.

Using averages from the ASA24 per time point, we estimated participants’ total energy intake, total protein and total carbohydrate intake, and diet components targeted by intervention (e.g., sugars, added sugars, sodium, fruits, vegetables, whole grains, fiber, solid fats, and saturated fats).

#### Dietary: Psychosocial and social support measures.

To assess self-reported eating behavior, participants completed the Three Factor Eating Questionnaire subscales^38^: cognitive restraint, uncontrolled eating, and emotional eating. Self-efficacy in healthy eating was measured using the 14 items from the Sallis Self-Efficacy for Diet Behaviors,^39^ resulting in the following subscales related to confidence in the ability to stick to low-saturated-fat and low salt foods, eat smaller portions, avoid adding salt at the table, and choose low-saturated-fat foods. The Sallis Social Support for Diet Behaviors^40^ is a 20-item survey with subscale scores for: family encourages healthy eating habits; family discourages healthy eating habits; friends encourage healthy eating habits, and friends discourage healthy eating habits. Attitudes toward healthy eating were measured using the Healthy Eating Attitudes Scale (HEA).^41^ All variables are scored in such a way that we expected the intervention to increase all these measures.

#### Physical activity. Aerobic activity and sedentary behavior.

At each time point, participants wore an ActiGraph Model GT3XE (ActiGraph LLC) accelerometer at the hip for 7 days, and only removed it when sleeping, bathing, or swimming. We implemented the Choi et al.^42^ algorithm to detect nonwear time and excluded days with < 10 hours of wear time. We categorized activity as light intensity physical activity and moderate to vigorous intensity physical activity using Freedson cut points.^43^ We assessed self-reported physical activity using the International Physical Activity Questionnaire-Short Form to metabolic equivalent (MET) minutes per week^44^ and sedentary behavior using the Sedentary Behavior Questionnaire.^45^

#### Physical activity: Strength training.

Participants completed functional fitness tests to measure overall strength and agility, upper and lower body strength, and endurance.^46^ The measures included an arm curl (number of arm curls with a 5-pound weight in 30 seconds), chair stand (number of stands in 30 seconds), 2-minute step test (number of midthigh height steps in 2 minutes), and 8-foot up and go (seconds to stand, walk 8 feet, walk back, and sit).

#### Physical activity: Psychosocial and social support.

We assessed self-efficacy for physical activity with 14 items from the Sallis Self-Efficacy for Exercise, resulting in subscales for self-efficacy for the following: sticking to exercise habits and for making time for exercise^39^; social support for physical activity with the 13 items from the Sallis Social Support for Exercise, with subscales for: family participation in exercise, friend participation in exercise, and family rewards and punishment for exercise^40^; and attitudes toward exercise with a 14-item scale, including questions such as (1) I should exercise more than I do and (2) I often feel guilty about my lack of exercise.^47^

### Statistical Analyses

#### Mediation analysis.

**Figure 1.**
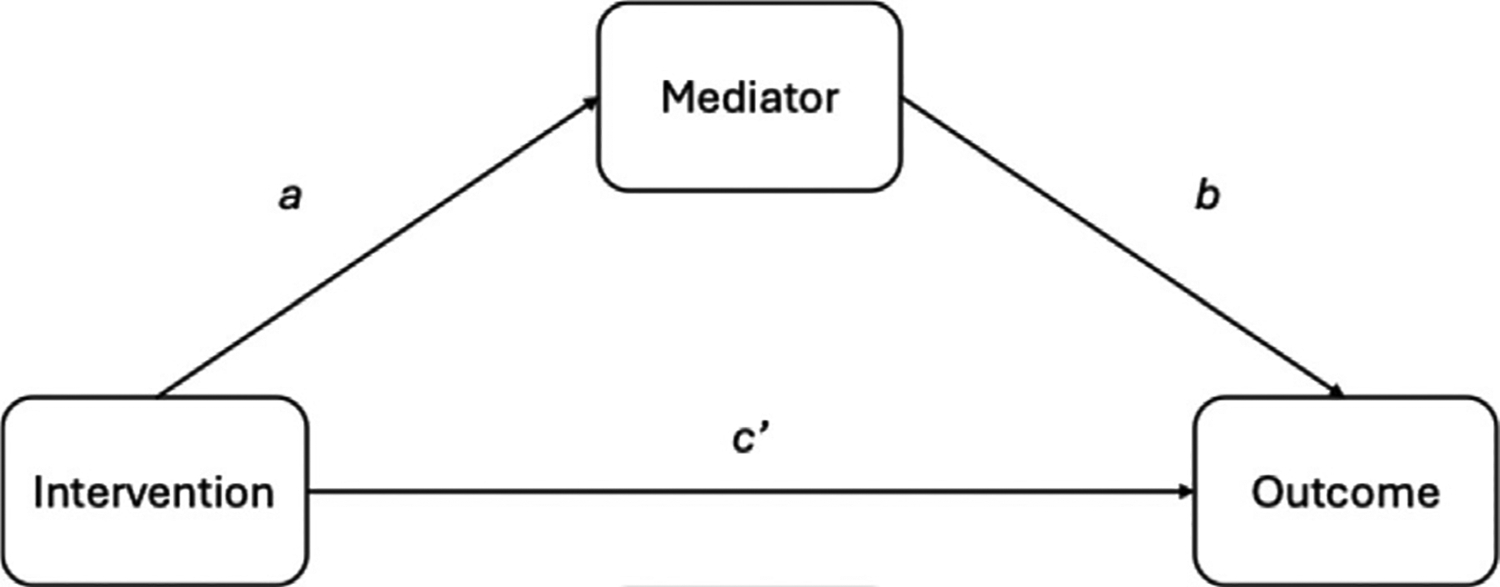
Representation of effects in the mediation analysis. This figure displays the pathways evaluated in the mediation analysis. a indicates the intervention effects on the mediator; b, the effect of change in the mediator on weight loss; c, represents the effect of the intervention on weight loss.

We conducted a traditional mediation analysis consisting of 2 independent mixed linear regressions to explore the potential effect of mediators on weight loss between intervention and comparison groups from baseline to the end of intervention (0 to 24 weeks). Pathway a is the regression of intervention effects on the mediator (a coefficient: LSE intervention effect), and pathway b is the regression of weight loss from the changes in mediator (b coefficient: LSE mediator effect) (Figure 1). Pathway c is the effect of the intervention on weight loss. While pathway c is the product of the 2 coefficients a × b, it represents the indirect effect of intervention on weight loss through the mediator, of which the asymptotic SE is as follows: square root (a^2^ × SE [b]^2^ + b^2^ × SE[a]^2^).^48,49^ We calculated the proportion mediated using the following formula: abs(ab)/(abs[ab] + abs[c’]) with consideration of the situation in which direct and indirect effects counteract one another.^50^ We used a 1-sided test to determine the significance of the mediator on weight loss at the *P* < 0.05 level. We chose to evaluate significance at the *P* < 0.05 level because of the exploratory nature of this analysis. While the intervention was meant to simultaneously target multiple mediators, we assessed only single-mediator models because of multicollinearity.^51^ Given our objective to elucidate the effect of the intervention rather than the intercluster differences, we reported statistics separately for the comparison and intervention groups. All models used complete case data and included random cluster (community) effects to account for the community-level randomization and correlation between participants in the same community. This model, using a blocked covariance matrix, effectively adjusted for potential unequal distributions, thereby enhancing the detection of significant explanatory variables. Covariates in the models, determined *a priori*, included age and education. We conducted intention-to-treat analyses that included all participants per random assignment, regardless of the number of assessments obtained or intervention attendance. To test the potential for erroneous conclusions based on data not missing at random, we conducted a tipping point analysis to determine the point at which values in missing data overturn the main findings.^52^

We conducted all analyses in 2022–2024 using SAS software (version 9.4, SAS Institute Inc., 2023) and R software (R Foundation for Statistical Computing, 2017). Dr Seguin-Fowler, as the study principal investigator, had full access to all study data and takes responsibility for its integrity and the data analysis.

## RESULTS

### Study Sample

**Figure 2.**
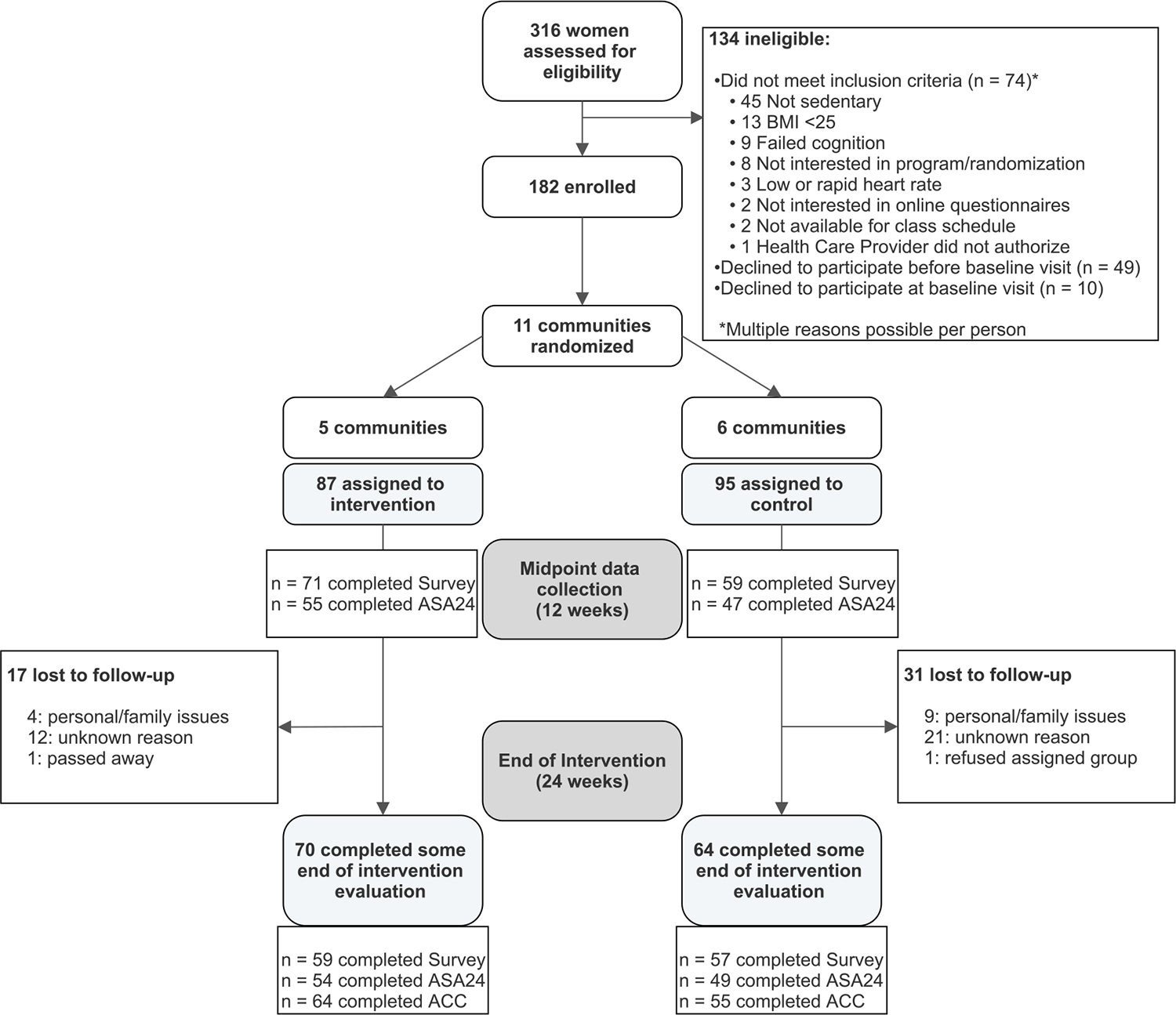
Participant flow through the Strong People, Strong Hearts-2.0 Trial. ACC indicates accelerometry; ASA24, automated self-administered 24-hour dietary recalls; BMI, body mass index.

We screened 316 individuals and enrolled 182 participants into the study (Table 2). 5 communities were randomized to the intervention group (n = 87 participants) and 6 communities to the delayed-intervention comparison group (n = 95 participants) (Figure 2). Class sizes ranged from 7 to 17 women. Over the period of 24 weeks, class attendance averaged 59% (67% in weeks 1–12; 52% in weeks 13–24).

### Missing Data

For the outcome variable of weight, no data were missing at baseline; 50 participants withdrew or did not complete the data collection visit (0% at baseline, 27% at 24 weeks). For mediators, missingness varied by data source: survey (5% at baseline, 37% at 24 weeks), accelerometry data (3% at baseline, 35% at 24 weeks), functional fitness test (0% at baseline, 29% at 24 weeks), and dietary recall data (18% at baseline, 43% at 24 weeks). The primary concern was that data may not be missing at random and that participants with worse health might be more likely to drop out or not report. To explore this potential bias, we compared baseline characteristics of respondents and nonrespondents at 24 weeks and observed no significant differences across baseline characteristics (age, income;, education, race, number of children, BMI, weight, meeting physical activity guidelines, or self-reported perceived overall health) for outcome measures, functional fitness test, accelerometry data, or dietary recall data. For the survey, nonrespondents tended to have a higher BMI at baseline compared with respondents. Furthermore, no differences in missing data rates were observed between the intervention and comparison groups.

### Mediation Analysis

Intervention effects on behaviors and psychosocial factors. We present the effect of the intervention on all potential mediators and the mediators’ effect on weight loss in Table 3. The SHHC-2.0 intervention improved diet quality as measured by the REAP-S (*P* < 0.001), and reduced energy intake (*P* = 0.02) as measured by the ASA24, along with reductions in fat, sugars, and sodium intake. Intervention participants demonstrated improved HEA (*P* = 0.01), uncontrolled eating (*P* = 0.001), and cognitive restraint (*P* < 0.001). Friends encouraging healthy eating also improved in response to the SHHC-2.0 intervention (*P* = 0.03). Both accelerometer-measured (moderate to vigorous intensity physical activity) and self-report (MET min/wk and vigorous intensity MET min/wk) measures of physical activity increased significantly in the intervention group; the intervention group demonstrated a decrease in attitudes toward exercise. Social factors (friends encouraging healthy eating and family participation in exercise) improved in the intervention group.

Mediation effects of behaviors and psychosocial factors on weight loss. In Table 4, we present the results of the mediation analysis for mediators that were both significantly impacted by the intervention and led to weight loss. Improvements in diet quality as measured by the REAP-S (*P* = 0.03), HEA (*P* = 0.04), and cognitive restraint (*P* = 0.01) had mediation effects on weight loss in the intervention group compared with the comparison group. Their contribution to the intervention effects was 31.8% for REAP-S, 23.0% for HEA, and 29.8% for cognitive restraint. For physical activity measures, the intervention group worsened in their attitudes toward exercise, and this was found to be a mediating variable to weight loss for the intervention group compared with the comparison group (*P* = 0.01), with a 22.6% contribution to the intervention effect.

## DISCUSSION

The role of dietary behavior change in weight loss is well-established.^53^ A large proportion of the SHHC-2.0 curriculum focuses on dietary behavior change. Participants are provided with recommended energy intake and taught to target that energy intake by tracking food portions across food categories. In the SHHC-2.0 curriculum, there is also a focus on changing dietary patterns—based on US dietary guidelines, DASH, and Mediterranean dietary patterns^28–31^—with an emphasis on increasing fruit and vegetable consumption, while reducing energy intake, sodium, saturated and trans fats, sugar-sweetened beverages. Decrease in energy intake was not identified as a mediator; however, that finding may reflect the challenges in accurately measuring energy intake through dietary recalls.^54^ Improvement in HEA was also identified as a mediator for weight loss, which aligns with attitudes as a determinant of behavior in Social Cognitive Theory^55^ and the Theory of Planned Behavior^56^ While the HEA scale is intended to broadly assess attitudes toward healthy eating, it is important to note that 1-item in the scale is “It is important that the food I eat helps me control my weight,” which may influence the size of the mediation effect observed.

As noted above, a dietary behavioral aim of SHHC-2.0 is focused on decreasing energy intake, and thus an effect on cognitive restraint was anticipated. Examples of topics within SHHC classes that align with cognitive restraint include controlling portion sizes, goal setting, menu planning, mindful eating, and a home food environment assessment. In addition, participants received their dietary restraint scores at baseline and midway through the intervention. The significant role of cognitive restraint in weight loss in the SHHC-2.0 trial is in line with previous research. Though originally conceptualized as risk for overconsumption and obesity,^57^ studies suggest increased cognitive restraint following behavioral weight loss interventions instead reflects a conscious restriction of food intake with the intent to lose weight. This is well-supported in the literature with consistent findings indicating that increased cognitive restraint is associated with greater weight loss in behavioral weight loss trials.^58,59^ Increased dietary restraint has also been shown to have a role in weight loss maintenance,^59,60^ suggesting that the effects of the SHHC-2.0 intervention may support maintenance of the weight loss experienced during the intervention period. Further supporting this notion, intervention participants’ weight loss was observed to remain significant 24 weeks after the intervention ended.^21^

This analysis indicates that the observed increases in physical activity did not significantly affect weight loss. These results align with existing evidence. While combined dietary and exercise interventions have been found to result in greater weight loss than dietary interventions alone, it is estimated that only 20% of the observed weight loss can be attributed to physical activity behavior.^61^ It is important to note that weight loss is only one measure of health improvement. The observed increase in physical activity because of SHHC-2.0^21,22^ has other potential positive health impacts, as increasing physical activity independent of weight has been shown to reduce all-cause mortality and risk of noncommunicable diseases, such as cardiovascular disease, type 2 diabetes, and certain cancers.^62^ Furthermore, while physical activity contributes minimally to initial weight loss, physical activity has been shown to support improved weight loss maintenance.^60^

Participants in the intervention group experienced diminished attitudes toward exercise, which was found to mediate the intervention effects on weight loss. On the surface, this finding seems to be contradictory to the observed increase in physical activity in the intervention group. However, the exercise attitudes scale included the following questions: I should exercise more than I do, and I often feel guilty about my lack of exercise.^47^ It is possible that despite an increase in physical activity, the educational portions of the intervention resulted in increased knowledge of the benefits of physical activity and the physical activity guidelines, thus creating greater awareness and expectations around the amount of physical activity participants believed they should be engaging in.

In their review of weight loss interventions conducted in rural settings, Dixon et al.^4^ noted that 5 of the 18 interventions included in their review focused on factors beyond individual behaviors and called for more multilevel interventions to address rural/urban obesity disparities. SHHC-2.0 was a multilevel intervention that targeted involvement of friends and family in behavior change. We observed an increase in family participation in exercise; however, social support for dietary behaviors was not directly improved through the SHHC-2.0 intervention, nor was social support for diet or physical activity a significant mediator of weight loss. While social support is generally hypothesized to improve weight loss and/or behavior change, evidence linking social support with improved weight loss outcomes is mixed. For example, Kiernan et al.^63^ identified 3 subgroups among participants in a behavioral weight loss intervention by which social support impacted weight loss. They found that women who never received family support were less likely to achieve clinically-significant weight loss, while those who received frequent friend and family support were more likely to achieve significant weight loss. Contrary to the hypothesis, those who never received social support from friends were also more likely to achieve significant weight loss. This suggests that the types of social support received, and individual needs of each participant, are likely to impact the effect of social support on weight loss and help to explain the equivocal data to date^64,65^ In our trial, measures asked about friend and family support and may not have captured social support received from other participants or SHHC-2.0 leaders that may affect weight loss.

The results of this analysis should be interpreted with consideration of the limitations of our study. Most SHHC-2.0 participants were white, which is reflective of the racial/ethnic diversity within the rural New York towns from which participants were recruited. However, the demographic composition of rural communities across the US varies greatly. Interpretation of our results should also factor in that the study was not powered to test mediators on the primary outcome of weight. Furthermore, *a priori* selection of potential mediators for evaluation was focused on factors related to behavioral aims of the SHHC 2.0 intervention. It is possible that additional social or environmental factors could also mediate the intervention effects. Similarly, physiological mediators were not assessed in this study but could further inform intervention effects. For example, as noted in the introduction, hormonal changes among midlife women may affect body weight. While we would not anticipate the intervention to have a direct effect on these hormonal markers, it is possible that changes in these factors that are naturally occurring with age could impact the effects of the intervention. The combination of a relatively small sample size to test mediation, along with missing data and suboptimal adherence rates, may have also reduced the effect size observed in our analysis; as such, our findings should be interpreted as exploratory. To test the plausibility of erroneous conclusions based on missing data, a tipping point sensitivity analysis was conducted. We found that those lost to follow-up in the intervention group would have to decrease their REAP score by 8 points or more (on a scale of 13–39) than the comparison group during the 24-week intervention period to reverse the findings. We also ran multiple imputation models for this analysis that yielded the same findings (not reported), but report the complete cases estimates for consistency with previous analyses. Finally, we chose not to run multivariate mediation models because of the exploratory nature of this analysis and issues of multicollinearity. Therefore, the percent of contribution of the intervention through mediator (REAP, 31.8%; HEA, 23.0%; cognitive restraint, 29.8%; and attitudes toward exercise, 22.6%) should not be viewed as cumulative but rather that these variables are likely interrelated.

## IMPLICATIONS FOR RESEARCH, PRACTICE, AND POLICY

Our findings highlight relevant behavioral intervention targets (HEA and cognitive restraint) associated with weight loss and thus could inform both research and practice to ensure that interventions that are developed and/or delivered in practical settings are engaging these targets. From a practical perspective, the participant attendance and missing rates highlight potential challenges in intervention implementation and suggest the need to identify additional strategies (i.e., flexible scheduling, hybrid intervention options, and child care) to support intervention attendance that may be particularly relevant for persons living in rural areas. Results from a process evaluation conducted after the intervention support this notion.^27^ Participants reported several barriers to attendance, including lack of transportation, long travel times (> 1 hour), and additional responsibilities (i.e., child’s school, second job). Our findings build on previous research demonstrating the potential of the SHHC-2.0 intervention to improve health outcomes^20–23^ and provide insight into further SHHC-2.0 intervention refinements or new intervention development for improving cardiovascular disease risk through dietary and physical activity behavior change. Our findings also highlight potential areas for future research and intervention development. As noted above, the sample of our study was racially homogeneous (97.6% non-Hispanic White), indicating the need for replication in more diverse populations. Findings from these future studies may then indicate the need for further tailoring for specific rural populations. We did not observe an intervention effect on social support or an effect of social support on weight loss. Our findings suggest the need for additional strategies to build social support related to dietary behavior change. Social support is an element of the environment that influences behavior in the Social Cognitive Theory. It is also possible that building social support could result in improved intervention adherence, as greater social support is associated with greater engagement in health promotion programs among women living in rural communities.^66^ One potential strategy for improving social support could be the engagement of friends and/or family in the intervention. Previous research has demonstrated that participating in a behavior change program with friends and family has been found to result in greater weight loss compared with participating alone.^67^ The lack of increased social support observed in our study may also reflect unique challenges to fostering social support in rural settings that were not addressed by the intervention. As such, future work could aim to identify effective strategies to build social support among persons living in rural settings. In addition, it is possible that the measure of social support did not fully capture social support gained from intervention leaders or other participants, a consideration that may be important in future assessment of social support in group behavior change interventions. We did not evaluate environmental factors as potential mediators. These factors are likely to be slow to change and require future research with longer follow-up periods to assess the impact of environmental change on weight loss outcomes. Finally, because of its exploratory nature, we limited our analysis to single-mediator models. Given the potential complex interrelationships between psychosocial factors, future research can aim to explore multiple mediator models.

## Figures and Tables

**Table 1.** Strong Hearts, Healthy Communities-2.0 Program Overall Goals and Behavioral Aims and Associated Hypothesized Mediators

Variables	Hypothesized Intervention Effect (Data Source)
Overall goals	
Improve dietary patterns	Increase Healthy Eating Index total score (ASA24)
	Increase HEI component scores (ASA24)
	Increase Rapid Eating Assessment for Participant score (survey)
	Increase healthy eating attitudes (survey)
	Decrease uncontrolled eating score (survey)
	Increase cognitive restraint (survey)
	Decrease emotional eating (survey)
	Increase dietary self-efficacy (survey)
	Increase dietary social support (survey)
Be as physically active as possible every day	Increase light intensity physical activity (accelerometer)
	Increase moderate to vigorous intensity physical activity (accelerometer)
	Increase IPAQ total MET minutes (survey)
	Increase IPAQ moderate intensity MET minutes (survey)
	Increase IPAQ vigorous intensity MET minutes (survey)
	Increase IPAQ walking MET minutes (survey)
	Increase attitudes toward exercise (survey)
	Increase physical activity self-efficacy (survey)
	Increase physical activity social support (survey)
Behavioral aims	
Increase fruits and vegetables	Increase fruit servings (ASA24)
	Increase vegetable servings (ASA24)
Increase whole grains	Increase whole grains (ASA24)
	Increase fiber (ASA24)
Decrease energy	Decrease energy (ASA24)
Decrease desserts	Decrease added sugar (ASA24)
Decreased processed foods	Decrease percentage of energy from ultraprocessed foods (ASA24)
Decrease saturated and trans fats	Decrease saturated fat (ASA24)
	Decrease solid fat (ASA24)
Decrease sodium	Decrease sodium (ASA24)
Decrease SSBs	Decrease added sugar (ASA24)
Increase strength training to ≥ 2 times/wk	Increase overall strength and agility (FFT: 2-min step test and 8-ft up and go test)
	Increase lower body strength (FFT: chair stand test)
	Increase body strength (FFT: arm curl test)
Increase aerobic exercise to ≥ 5 times/wk	Increase moderate to vigorous intensity physical activity (accelerometer)
	Increase moderate intensity MET (IPAQ)
	Increase vigorous intensity MET (IPAQ)
Minimize sedentary time	Decrease total sitting hours per week (SBQ)

ASA24 indicates automated self-administered 24-hour dietary recalls; FFT, functional fitness test; HEI, Healthy Eating Index; IPAQ, International Physical Activity Questionnaire-Short Form; MET, metabolic equivalent; MVPA, Moderate to Vigorous Physical Activity; SBQ, Sedentary Behavior Questionnaire; SSB, sugar-sweetened beverage.

**Table 2.** Strong Hearts, Healthy Communities-2.0 Participant Characteristics at Baseline

Variables	Total	Control	Intervention	P
Participants, n (%)	182 (100)	95 (52.2)	87 (47.8)	
Age, y, mean ± SD	57.2 ± 9.0	55.9 ± 8.5	58.5 ± 9.3	0.53
Race/ethnicity (n = 168), n (%)				0.96
White, non-Hispanic	164 (97.6)	84 (97.7)	80 (97.6)	
Non-White or Hispanic	4 (2.4)	2 (2.3)	2 (2.4)	
Income (n = 162), n (%)				0.24
< $25,000	29 (17.9)	17 (20.0)	12 (15.6)	
$25,000–50,000	37 (22.8)	15 (17.6)	22 (28.6)	
> $50,000	96 (59.3)	53 (62.4)	43 (55.8)	
Relationship status (n = 171), n (%)				0.33
In a relationship	116 (67.8)	62 (71.3)	54 (64.3)	
Not in a relationship	55 (32.2)	25 (28.7)	30 (35.7)	
Education (n = 172), n (%)				0.94
High school or less	26 (15.1)	14 (16.1)	12 (14.1)	
Some college/technical or vocational school	35 (20.3)	17 (19.5)	18 (21.2)	
College graduate	63 (36.6)	33 (37.9)	30 (35.3)	
Postgrad/professional	48 (27.9)	23 (26.4)	25 (29.4)	
Smoking (n = 171), n (%)				0.84
Never	100 (58.5)	49 (56.3)	51 (60.7)	
Former	69 (40.4)	37 (42.5)	32 (38.1)	
Current	2 (1.2)	1 (1.2)	1 (1.2)	
Overall health (n = 175), n (%)				0.41
Excellent/very good	46 (26.3)	20 (22.5)	26 (30.3)	
Good	99 (56.6)	50 (56.2)	49 (57.0)	
Fair/poor	30 (17.1)	19 (21.3)	11 (12.7)	
Body mass index (n = 182), mean ± SD	36.7 ± 7.8	37.9 ± 8.5	35.4 ± 6.8	0.02
Self-report condition/disease (n = 170), n (%)				
High blood cholesterol	71 (41.8)	33 (38.4)	38 (45.2)	0.22
Hypertension	71 (41.8)	41 (47.7)	30 (35.7)	0.23
Arthritis	70 (41.2)	39 (44.8)	31 (37.3)	0.45
High blood sugar	37 (21.8)	16 (19.3)	21 (24.1)	0.22
Diabetes	25 (14.7)	17 (19.5)	8 (9.6)	0.09
Cancer	12 (7.1)	5 (5.7)	7 (8.4)	0.45
Heart disease	10 (5.9)	5 (5.7)	5 (6.0)	0.24
Kidney disease	3 (1.8)	1 (1.1)	2 (2.4)	0.51

Note: Differences in means for continuous variables were evaluated using t tests. Differences in percentages for categorical variables were evaluated using chi-square tests.

**Table 3.** Estimated Effect of Strong Hearts, Healthy Communities-2.0 intervention on Potential Mediators and Effect of Potential Mediators on Weight Loss

		Effect of Interventionon Mediator			Effect of Mediatoron Weight Loss		
Overall Dietary Patterns	n	Estimate	95% CI	*P*	Estimate	95% CI	*P*
HEI score (100 pts) (ASA24)	97	4.37	−0.34 to 9.08	0.07	−0.08	−0.16to0.00	0.05
HEI component–total fruits (5 points)	97	0.23	−0.48 to 0.94	0.53	−0.62	−1.05 to −0.20	0.01
HEI component–whole fruits (5 points)	97	0.19	−0.61 to 0.99	0.64	−0.42	−0.80 to −0.04	0.04
HEI component–total vegetables (5 points)	97	0.37	−0.15to0.89	0.17	0.21	−0.38 to 0.81	0.72
HEI component–greens and beans (5 points)	97	0.61	−0.28 to 1.51	0.18	−0.04	−0.39 to 0.30	0.42
HEI component–whole grains (10 points)	97	1.34	0.00 to 2.69	0.05	0.06	−0.17to0.30	0.67
HEI component–dairy (10 points)	97	0.31	−0.83 to 1.44	0.60	−0.03	−0.30 to 0.25	0.43
HEI component–total protein foods (5 points)	97	−0.06	−0.36 to 0.24	0.70	−0.12	− 1.16to0.92	0.42
HEI component–seafood and plant proteins (5 points)	97	0.72	−0.29 to 1.74	0.17	−0.21	−0.51 to 0.09	0.13
HEI component–fatty acids (10 points)	97	0.33	− 1.09 to 1.75	0.65	−0.1	−0.32 to 0.12	0.23
HEI component–refined grains (10 points)	97	0.02	− 1.24 to 1.28	0.97	−0.35	−0.59 to −0.11	0.01
HEI component–sodium (10 points)	97	−1.14	−2.17to −0.10	0.03	−0.03	−0.33 to 0.27	0.43
HEI component–added sugar (10 points)	97	0.79	0.07–1.52	0.04	0.01	−0.42 to 0.43	0.51
HEI component–saturated fat (10 points)	97	0.64	−0.62 to 1.91	0.32	−0.17	−0.41 to 0.08	0.13
Rapid Eating Assessment for Participants score (scale 13–39) (survey)	115	3.54	2.12–4.97	< 0.001	−0.30	−0.56 to −0.04	0.03
Percentage of calories from ultraprocessed foods (ASA24)	105	−1.11	−7.39 to 5.16	0.73	0.00	−0.06 to 0.06	0.96
Whole grains (oz) (ASA24)	97	0.23	−0.15to0.60	0.23	0.35	−0.64 to 1.34	0.49
Fruit (cup) (ASA24)	97	0.08	−0.29 to 0.46	0.66	−0.52	− 1.52 to 0.47	0.30
Vegetables (cup) (ASA24)	97	0.03	−0.32 to 0.38	0.87	0.15	−0.90 to 1.2	0.79
Nutrient intake (ASA24)							
Energy (kcal)	97	−229.14	−413.81 to −44.47	0.02	0.00	0.00–0.00	0.23
Protein (g)	97	−8.08	− 18.00 to 1.84	0.11	0.00	−0.04 to 0.04	1.00
Total fat (g)	97	−10.61	−20.54 to −0.68	0.04	0.01	−0.02 to 0.05	0.46
Solid fat (g)	97	−4.15	− 11.11 to 2.82	0.25	0.03	−0.02 to 0.09	0.24
Saturated fat (g)	97	−3.88	−7.86 to 0.09	0.06	0.05	−0.04 to 0.14	0.30
Carbohydrates (g)	97	−21.81	−44.61 to 0.99	0.06	0.01	0.00–0.03	0.13
Sugar (g)	97	−14.26	−27.24 to −1.29	0.03	0.01	−0.02 to 0.04	0.62
Added sugar (g)	97	−3.17	−5.67 to −0.66	0.02	0.09	−0.05 to 0.24	0.22
Fiber (g)	97	1.33	−0.86 to 3.52	0.24	0.01	−0.16to0.18	0.89
Sodium (mg)	97	−165.92	−509.86 to 178.02	0.35	0.00	0.00–0.00	0.38
Diet psychosocial (survey)							
Healthy Eating Attitudes (scale 1–10)	114	0.27	0.06–0.48	0.01	−2.71	−4.50 to −0.93	0.004
Uncontrolled eating score (scale 6–24)	115	−2.36	−3.71 to −1.00	0.001	0.22	− 0.05 to 0.50	0.11
Cognitive restraint score (scale 6–24)	115	2.08	0.95–3.21	< 0.001	−0.47	−0.80 to −0.14	0.006
Emotional eating score (scale 6–24)	115	−0.75	− 1.52 to 0.02	0.06	0.38	−0.10to0.86	0.12
Self-efficacy: Stick to low-saturated-fat, low salt foods (scale 1–5)	115	0.13	−0.29 to 0.55	0.55	−0.29	− 1.23 to 0.65	0.55
Self-efficacy: Eatsmaller portions (scale 1–5)	115	−0.18	−0.56 to 0.19	0.34	−0.28	− 1.33 to 0.77	0.61
Self-efficacy: Avoid adding salt at the table (scale 1–5)	114	−0.17	−0.59 to 0.26	0.44	−0.36	− 1.28 to 0.55	0.44
Self-efficacy: Choose low-saturated-fat foods (scale 1–5)	115	0.10	−0.20 to 0.40	0.52	−0.77	−2.03 to 0.50	0.24
Social support: Family encouraged healthy eating habits (scale 5–25)	7	0.54	− 1.63 to 2.72	0.63	−0.13	−0.32 to 0.07	0.20
Social support: Family discouraged healthy eating habits (scale 5–25)	74	−0.48	−2.22, to 1.26	0.59	0.17	−0.11 to 0.45	0.25
Social support: Friends encouraged healthy eating habits (scale 5–25)	78	2.42	0.25–4.59	0.03	−0.22	−0.42 to −0.02	0.03
Social support: Friends discouraged healthy eating habits (scale 5–25)	57	0.20	− 1.72 to 2.12	0.84	−0.10	− 0.59 to 0.40	0.71
Physical activity levels							
Average light intensity physical activity (min/d) (accelerometer)	118	−4.30	−25.36 to 16.77	0.69	0.01	−0.01 to 0.03	0.29
Average moderate to vigorous intensity physical activity	119	6.57	1.67–11.47	0.01	−0.06	−0.13to0.02	0.14
(min/d) (accelerometer)							
IPAQ total MET min/wk (survey)	114	936.14	227.50–1,644.77	0.01	0.00	0.00–0.00	0.15
IPAQ walking MET min/wk (survey)	114	239.4	−39.43 to 518.22	0.10	0.00	0.00–0.00	0.19
IPAQ moderate intensity MET min/wk (survey)	114	241.19	−62.07 to 544.45	0.12	0.00	0.00–0.00	0.52
IPAQvigorous intensity MET min/wk (survey)	114	455.55	64.60–846.50	0.02	0.00	0.00–0.00	0.23
SBQ: Total sitting h/wk (survey)	112	1.06	−7.80 to 9.93	0.81	0.00	− 0.05 to 0.04	0.85
Functional fitness measures (n = 182), mean ± SD							
Chairstand, no. of stands	130	3.52	2.34–4.69	< 0.001	−0.13	−0.41 to 0.15	0.36
8-ft up and go, s	131	−0.42	−0.78 to −0.07	0.02	0.55	−0.37 to 1.47	0.24
Arm curl, no. of curls	131	4.53	2.83–6.22	< 0.001	0.08	−0.11 to 0.27	0.40
2-min step test, no. of steps	129	14.28	6.79–21.76	< 0.001	−0.03	−0.08 to 0.01	0.14
Physical activity psychosocial (survey)							
Self-efficacy: sticking to exercise habits (scale 1–5)	114	0.18	−0.18to0.55	0.33	−0.86	− 1.93 to 0.20	0.11
Self-efficacy: making time for exercise (scale 1–5)	114	0.14	−0.25 to 0.52	0.49	−0.63	− 1.65 to 0.39	0.23
Social support: Family participation in exercise (scale 10–50)	95	5.71	2.60–8.82	0.001	−0.02	−0.14to0.09	0.71
Social support: Friend participation in exercise (scale 10–50)	91	3.58	−0.16to7.33	0.06	−0.05	−0.20 to 0.09	0.45
Social support: Family rewards and punishment for exercise (scale 3–15)	94	0.1	−0.52 to 0.72	0.76	−0.06	− 0.65 to 0.53	0.84
Combined attitudetoward exercise score (scale 1–14)	115	−0.2	−0.34 to −0.06	0.005	3.77	1.06–6.49	0.008

ASA24 indicates automated self-administered 24-hour dietary recalls; IPAQ, International Physical Activity Questionnaire; MET, metabolic equivalent; NCI FV Screener, National Cancer Institute Fruit and Vegetable Screener; SBQ, Sedentary Behavior Questionnaire.

**Table 4.** Estimated effect of intervention on weight loss through the mediator and percent contribution of mediator on weight loss

	Estimated weight loss in kg	95%CI	p-value	Contribution of mediator
Rapid Eating Assessment for Participants score (scale 13 to 39)	−1.05	(−1.91,−0.20)	0.026	31.8%
Healthy Eating Attitudes (scale 1–10)	−0.74	(−1.37,−0.11)	0.036	23.0%
Cognitive restraint score (scale 6–24)	−0.76	(−1.70,−0.25)	0.006	29.8%
Friends encouraged healthy eating habits	−0.54	(−1.11, 0.03)	0.061	-
Combined attitude toward exercise score (scale 1–14)	−0.76	(−1.39,−0.12)	0.008	22.6%

All estimates are from complete case models and adjusted for random cluster (community) effects, random assignment group, age, and education. Confidence intervals and p-value adjusted for one-side test.

95%CI= 95% Confidence interval.
